# Status of US Emergency Medical Service Protocols Regarding Pre-Transfer Cooling for Exertional Heat Stroke

**DOI:** 10.7759/cureus.19505

**Published:** 2021-11-12

**Authors:** Aaron J Monseau, Gage A Hurlburt, Brenden J Balcik, Kathryn E Oppenlander, Nicholas M Chill, Peter S Martin

**Affiliations:** 1 Emergency Medicine/Sports Medicine, West Virginia University, Morgantown, USA; 2 Medicine, West Virginia School of Osteopathic Medicine, Lewisburg, USA; 3 Family Medicine, Carl R. Darnall Army Medical Center, Fort Hood, USA; 4 Emergency Medicine, West Virginia University School of Medicine, Morgantown, USA

**Keywords:** cold water immersion, emergency medical services, protocol, cooling, heat illness, exertional heat stroke

## Abstract

Objective: Exertional heat stroke (EHS) is a significant cause of morbidity and mortality in athletes and active individuals. In the field, initial management of exertional heat illness is based on rapid whole-body cooling. Cold-water immersion (CWI) is considered the superior cooling modality for EHS treatment. However, there often is a disconnect between the sports medicine community and the emergency medical service (EMS) community. Well-written emergency action plans may fail if EMS protocols do not allow for CWI in initial management. This is the first study to look at the current national EMS protocols regarding prehospital management of EHS. The purpose of our study was to assess the status of heat illness protocols regarding CWI for EHS in all 50 states plus Washington, DC.

Methods: An internet search was performed to find EHS protocols. Statewide protocols were preferred. Several parameters were recorded for each protocol including whether: 1) CWI was the recommended cooling treatment for EHS and 2) CWI was explicitly permitted to be completed prior to transportation.

Results: We found nine of the 51 protocols, or 17.6%, explicitly recommended CWI and 11 of the 51, or 21.6%, specifically instructed EMS personnel to complete CWI or cooling methods prior to transport. However, six protocols, or 11.8%, provided the recommendation instructing some variation of the phrase “do not delay transport to cool the patient.”

Conclusion: Despite the medical literature endorsing CWI as the most effective treatment modality in a prehospital setting for exertional heat illness, EMS protocols largely fail to reflect this which leads to mismanagement and inadequate care of EHS patients. While CWI is not always available, all EMS protocols should include a systematic practical guideline for a heat illness patient when employing cooling treatment with an emphasis on CWI when available as the preferred treatment technique for EHS and the concept of “cool first, transport second.”

## Introduction

Exertional heat stroke (EHS) is a unique form of heat illness which is life-threatening and may occur in any individual who undergoes exertion beyond their physical limits or in an unfavorable environment. Heat illness is a rapid rise in body temperature, generally the result of the body’s impaired ability to dissipate heat at a rate that matches internal heat production [1]. EHS is defined as a core temperature >40˚C (104˚F) and central nervous system dysfunction (e.g. altered mental status, confusion, irritability, seizure) [1-4]. The risk of heat illness exists at any level of physical activity and environmental condition, typically during strenuous or prolonged exercise in hot or humid conditions but can also occur in a cool environment [2,5,6]. Predisposing factors that affect the body’s thermoregulatory system include dehydration, poor acclimatization, low physical fitness level, sleep deprivation, obesity, certain medications, recent illness, and excess clothing or equipment [1,2,4].

Immediate recognition and rapid cooling of EHS patients is critical for patient survival [7-9]. This requires a core body temperature measurement as soon as possible after the EHS presents, and it must not be impacted by external factors (e.g. sweat, fluid, wind, clothing, etc.). A rectal temperature is the clinical gold standard for establishing a proper diagnosis [2,3,5]. Other means of obtaining temperature (e.g. oral, temporal, axillary, skin) are unreliable, as these methods inaccurately measure an exercising individual’s internal body temperature, and thereby may lead to misdiagnosis or inadequate treatment [1-3,5]. According to the National Athletic Trainers’ Association (NATA) current position statement on exertional heat illnesses, it is recommended that all patients with suspected EHS should obtain a rectal temperature assessment [5]. Medical staff should not risk wasting valuable time by utilizing invalid temperature methods or neglecting other key diagnostic indicators (e.g. mental status changes, collapse). In the event that EHS is suspected but no rectal thermometer is available, treatment with cold-water immersion (CWI) should still be initiated on scene if it is available [3,6].

Even with prompt recognition, immediate whole-body cooling is an essential component of EHS care since the major determinant of EHS outcome and degree of tissue injury is the duration of severe hyperthermia [2,5,9-11]. Therefore, CWI or other rapid cooling procedures are the primary treatment to immediately lower core body temperature [1-11]. CWI is best performed using a large tub with ice and water with continuous movement of the water, and a full description of the procedure can be found on the Korey Stringer Institute website. This remains the foundation of treatment of EHS patients as current literature recognizes a 100% survival rate when adequate cooling begins within 30 minutes of the first signs of struggling which makes the lasting sequelae of EHS such as end-organ damage or death entirely preventable with prompt recognition and emergent cooling [1,5,8]. CWI has shown superior cooling rates for treatment of EHS compared to other cooling methods and is the most effective treatment for minimizing morbidity and mortality. Patient cooling is recommended to be completed on-site prior to emergency transport [1-3,7]. The concept, “cool first, transport second” highlights the premise of EHS management, but the concern is that emergency medical service (EMS) providers may attempt to halt CWI that is previously in progress if their protocols do not allow for on-scene cooling. Furthermore, there is wide variability in EHS management by EMS providers because their protocols do not explicitly recommend CWI or do not allow CWI to continue if started before EMS arrival on scene [7].

The consensus standard of care for EHS involves a rectal temperature assessment and rapid pre-transfer cooling preferably by CWI [2,5,7]. However, medical personnel may be limited at the scene due to a lack of cooling equipment or supplies; furthermore, poor EMS protocols may create more barriers by failing to recommend on-scene cooling. When the protocol does not sufficiently address EHS treatment, the EMS responders must either take control of the scene and follow their protocol or contact their medical command physician (MCP) who then makes real-time decisions for onsite patient care. While the MCP may make the appropriate decision to initiate CWI, an appropriately written EMS protocol will obviate the need for a MCP call in the first place and reduce human error as the cause for inappropriate patient care. The EMS protocol for EHS needs to be comprehensive enough to instruct the on-scene provider to initiate CWI if it is available and permit CWI to continue if it has already started. A well-written protocol will avoid necessitating a MCP call in a CWI situation and therefore prevent the possibility of a management error. It is vital for cooling procedures to be initiated and completed prior to transport. When water immersion is not possible or cooling equipment is limited, other less effective cooling techniques are often utilized because of practicality and accessibility [1,7]. Sports Medicine physicians and EMS physicians have an opportunity to work together to improve the prehospital paradigm for patients suffering from EHS. While there may not be CWI available for the majority of EHS patients, it should be addressed in the EMS protocol to ensure that patients can receive CWI when it is available.

The purpose of our study was to assess the status of heat illness protocols regarding CWI for EHS in all 50 states plus Washington, DC. We suggest that every EMS protocol should specifically address CWI to both recommend it and explicitly permit it if already begun. In this study, we reviewed nationwide EMS heat-related illness protocols to outline the current model of care for EHS. Our hypothesis was that EMS protocols do not adequately state proper EHS management thus placing patients at high risk of poor outcomes and possible death.

## Materials and methods

**Figure 1:**
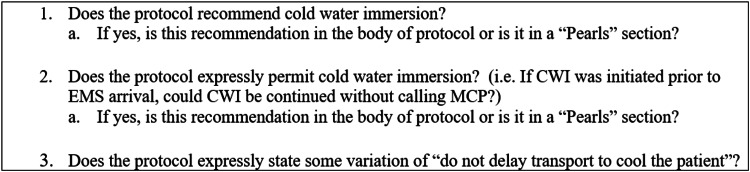
Specific questions utilized in the protocol review process. CWI=Cold Water Immersion, EMS=Emergency Medical Services, MCP=Medical Command Physician

After obtaining an IRB exemption from West Virginia University, Morgantown, we conducted a comprehensive search of United States EMS heat illness protocols and specifically addressed if it recommends or permits on-scene, pre-transfer CWI for EHS. This involved an internet search for EMS protocols deployed in all 50 states and District of Columbia. Our primary goal was to acquire a statewide EHS protocol if one existed. When there was no statewide protocol available, we identified a well-populated area in each state as our target locale. While we know that there are many high quality EMS Directors in rural locations, we felt that a well-populated area would have greater resources to devote to their EMS office which would increase the probability of having a higher quality set of protocols. Depending on the state, the non-statewide protocols were either regional, county, or city-based since each state has its own strategy for emergency medical management. Subsequently, two states did require direct email contact, as no known publicly available protocol was found. We recorded the type of protocol (i.e. state, regional, county, or city), whether CWI was recommended and/or permitted, where CWI was addressed in the protocol (i.e. body of the protocol, “pearls” or “notes” section, or in a separate protocol), and whether the incorrect recommendation of some variation of “do not delay transport to cool the patient” was made. Initial review of each protocol was performed by two authors (AJM and BJB). In the event that the two reviewers did not arrive at the same conclusion or were confused by a protocol, a third author (PSM) reviewed the protocol to adjudicate the finding. The specific questions that each reviewer attempted to answer for each protocol are listed in Figure 1. An additional search for supplemental EMS protocols indicating heat-related illness management and treatment was performed using keywords such as ice/water immersion, exertional heat, and sports.

A total of 51 EMS protocols were identified, and individual protocol information was recorded into Research Electronic Data Capture (REDCap, Vanderbilt University, Nashville, TN, USA). Study data were collected and managed using REDCap electronic data capture tools hosted at WVCTSI [12,13]. REDCap is a secure, web-based software platform designed to support data capture for research studies, providing 1) an intuitive interface for validated data capture; 2) audit trails for tracking data manipulation and export procedures; 3) automated export procedures for seamless data downloads to common statistical packages; 4) procedures for data integration and interoperability with external sources. Specifically, the REDCap survey was utilized to outline protocol details and export data related to prehospital care of heat illness.

## Results

**Figure 2:**
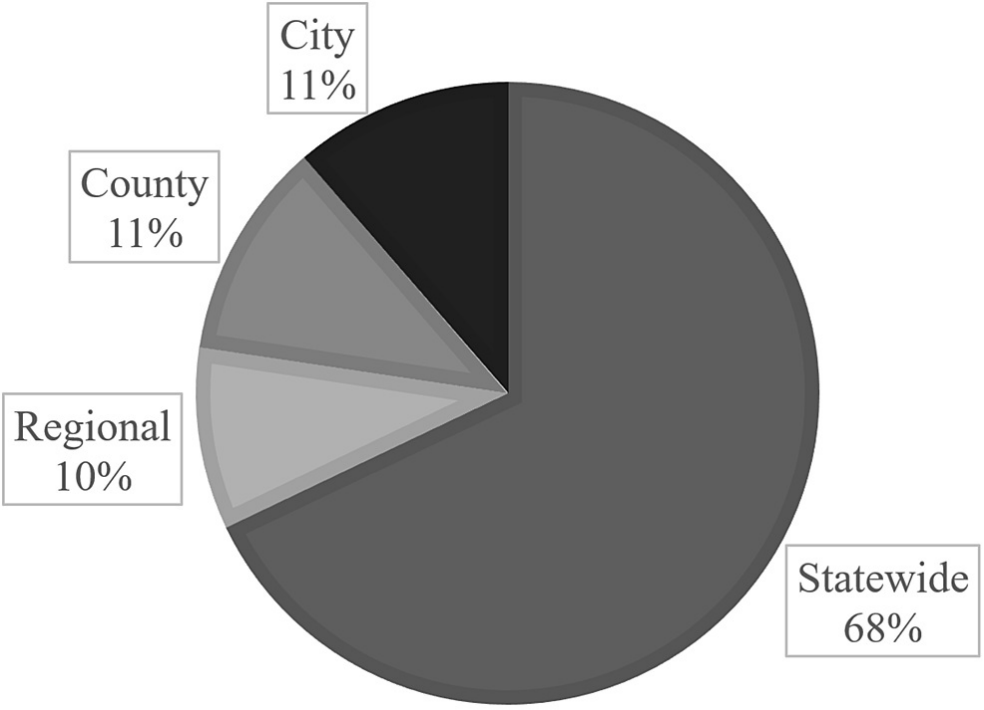
Proportion of EMS protocol type of the total selected protocol population. EMS=Emergency Medicine Services

A total of 51 protocols approved between the years 2005 and 2019 were selected in our study and specifically addressed a heat-related illness policy. Most states had a statewide EMS protocol with a total of 34 published protocol guidelines (Table 1). Otherwise, there were five regional protocols, six county protocols, and six city protocols (Figure 2).

**Table 1:** EMS protocols that included a heat-related policy for EHS. EMS=Emergency Medical Services, EHS=Exertional Heat Stroke

Protocol type	Number of protocols
Statewide	34
Regional	5
County	6
City	6

**Figure 3:**
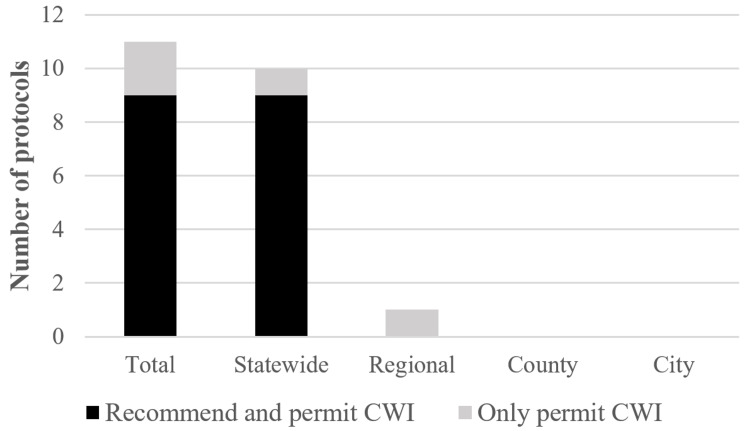
EMS protocols who recommended and permitted CWI to be completed once begun and prior to transportation, and those who permitted CWI but did not recommend it. EMS=Emergency Medical Services, CWI=Cold Water Immersion

We identified nine of the 51 protocols, or 17.6%, that recommended CWI which were all statewide protocols (Figure 3). Each of those nine protocols along with two additional protocols (one statewide and one regional) permitted CWI to be completed once begun which made 11 of 51, or 21.6%. Furthermore, six of the 11 protocols addressed CWI as part of the step-by-step algorithm, while four of the 11 addressed it in a separate “Pearls” section. Finally, one of 11 specifically discussed CWI in a separate protocol altogether, titled “Event Medicine.”

**Figure 4:**
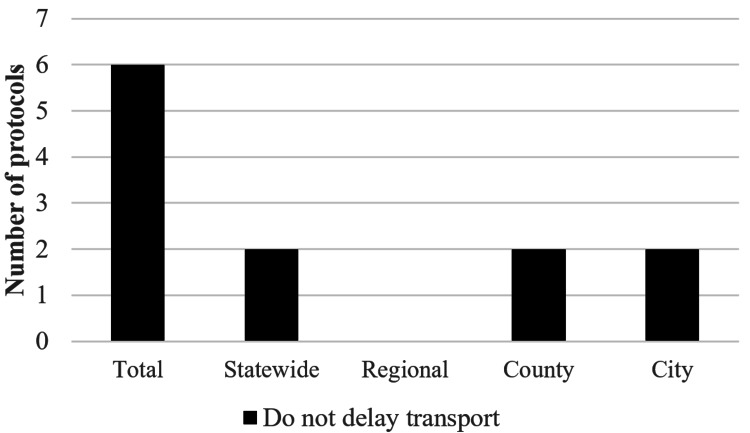
Incorrect EMS protocol recommendations regarding EHS. Figure depicts protocols who provided the incorrect recommendation of some variation of “do not delay transport to cool the patient.” EMS=Emergency Medical Services, EHS=Exertional Heat Stroke

**Figure 5:**
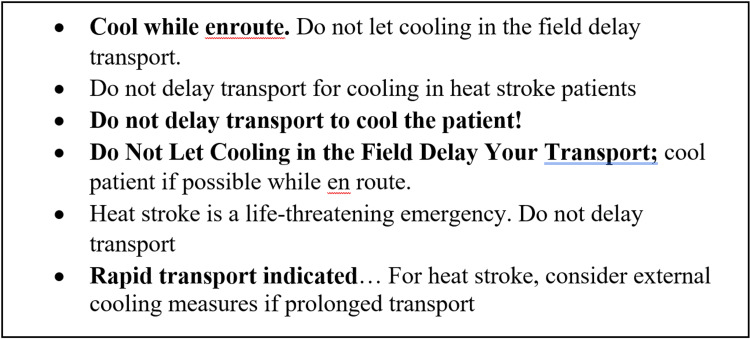
Exact wording, boldface, capitalization, and punctuation for the six protocols which recommend some variation of “do not delay transport to cool the patient”

**Figure 6:**
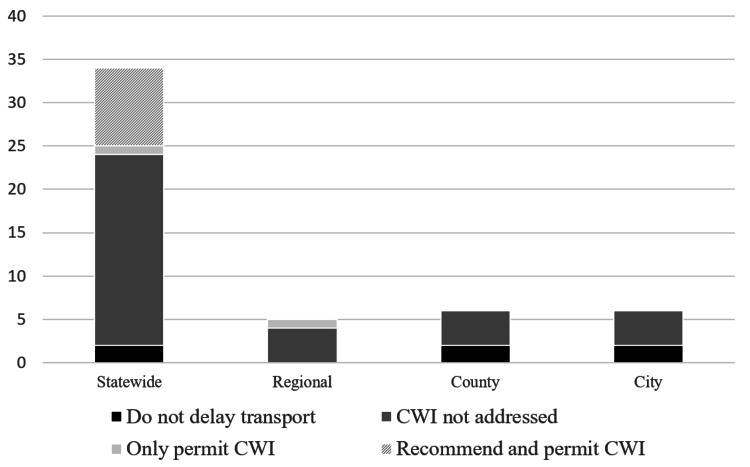
EMS protocol recommendations addressing CWI for EHS EMS=Emergency Medical Services, CWI=Cold Water Immersion, EHS=Exertional Heat Stroke

There were six protocols out of the 51, or 11.8%, that provided the incorrect recommendation instructing some variation of the phrase “do not delay transport to cool the patient”, and two of those five were statewide protocols (Figure 4). The exact wording of those six protocols can be found in Figure 5. By adding the 34 of 51 protocols where CWI was not addressed, this means that CWI was not correctly recommended in 40 of 51 protocols, or 78.4%. Figure 6 demonstrates the proportions of each type of protocol for each locale.

## Discussion

The purpose of this study was to investigate current recommendations for EHS pre-transfer cooling. Protocols addressing EHS management should be in accordance with best practices. We attempted to identify the number of protocols that recommended CWI as the preferred cooling modality and permitted onsite cooling completion prior to transportation. This was the first study to examine national EMS protocols regarding EHS treatment and revealed a low proportion of protocols recommending or even permitting the optimal treatment strategy. The findings showed that only nine, or 17.6%, of the studied protocols recommended CWI as the preferred cooling technique and 11, or 21.6%, permitted CWI or instructed to complete cooling once initiated.

It is apparent though, that most state EMS protocols do not correctly address CWI. Moreover, if the EMS crew were to initiate CWI without protocol reference, the EMS provider must obtain approval from the MCP. This often leads to confusion and inadequate onsite treatment, not to mention a delay in the initiation of treatment, which can significantly increase the probability of worse outcomes associated with the condition. While contacting the MCP may not seem significant, it introduces the element of human error into the patient’s care. This role is usually filled by an emergency physician on-shift at a hospital who has been contacted to provide this service. The emergency physician should have some working knowledge of the mandated EMS protocols and what is available to EMS providers, but he or she may have insufficient or no knowledge of out-of-hospital management for EHS when CWI is available. Even if the onsite medical staff (e.g. athletic trainer) advocates for the appropriate treatment with CWI, the EMS responders are not permitted to disobey the orders of the MCP. With a more intuitive protocol, it will improve care by preventing potential errors instructed by the MCP. While the ability to contact the MCP should be a part of every EMS protocol, EHS treatment should be described in a comprehensive enough way to avoid the need to involve the MCP.

Protocol placement is important to promote routine use in appropriate situations. A step-by-step algorithm in the appropriate heading is more effective than a separate appendix. Our results reveal six state-wide protocols with CWI discussed directly in the guideline algorithm, and future protocol revisions should emulate this. Other organizations including the American College of Sports Medicine and NATA also implement similar practical guidelines for EHS management [2,5]. All EMS protocols should include a systematic clinical pathway for EHS management with an emphasis on the concept “cool first, transport second” and utilization of superior cooling modalities (i.e. ice or CWI).

Furthermore, the revelation that five protocols give the recommendation instructing some variation of the phrase “do not delay transport to cool the patient” was shocking for our study team, and these should be revised as soon as possible. These findings demonstrate that appropriate emergency treatment plans are clearly not well represented in prehospital care for EHS. Current evidence endorses an important component of EHS care-stabilization and rapid cooling takes precedence over immediate transportation [1,2]. Even with substantial evidence supporting rapid, effective cooling, inadequate EMS recommendations leading to inconsistent onsite practices continue. Heat stroke emergency protocols must exclusively prioritize rapid cooling, specifically CWI when available, prior to transport as the standard of care. Other cooling methods which were proposed in protocols such as placing ice towels to the axilla and groin and directing a fan at the patient should be implemented when CWI is not available. More consistent and adequate prehospital EHS treatment enforced by EMS recommendations are a crucial step for minimizing patient morbidity and mortality.

Several limitations were inherent in this study. It is possible that protocols were updated after we captured them early in 2019. There is a chance that local protocols may be different than statewide protocols when EMS companies choose to vary from their state recommendations. Finally, the non-state protocols may not reflect the entire state since other areas in the state may have a more sufficient protocol that correctly addresses CWI. However, considering only the 34 statewide protocols, nine of those, or 26%, recommended CWI and 10, or 29%, permitted it once begun prior to transport. There were two statewide protocols, or 5.9%, that gave the incorrect recommendation of some variation of “do not delay transport to cool the patient” which leaves 22 statewide protocols, or 65%, that did not address CWI at all. 

## Conclusions

The medical literature endorses readily available, prehospital CWI as the most effective treatment modality for EHS. Unfortunately, EMS protocols from around the US largely fail to reflect this which may lead to mismanagement and inadequate care of EHS patients. In the statewide subset of EMS protocols alone, only a quarter recommended CWI. All EMS protocols should include a systematic, practical guideline for heat illness patients when employing cooling treatment with an emphasis on CWI as the preferred technique for EHS. Furthermore, every EMS protocol on heat illness should be reviewed to ensure that transport is adequately addressed so, when CWI is available, EHS patients can receive it with the advice to “cool first, transport second.”
